# 2,4-Dichloro-*N*-(2-methyl­phen­yl)benzene­sulfonamide

**DOI:** 10.1107/S1600536810035166

**Published:** 2010-09-11

**Authors:** B. Thimme Gowda, Sabine Foro, P. G. Nirmala, Hartmut Fuess

**Affiliations:** a Department of Chemistry, Mangalore University, Mangalagangotri 574 199, Mangalore, India; b Institute of Materials Science, Darmstadt University of Technology, Petersenstrasse 23, D-64287 Darmstadt, Germany

## Abstract

In the title compound, C_13_H_11_Cl_2_NO_2_S, the methyl-substituted aromatic ring is disordered over two positions [occupancy ratio 0.705 (5):0.295 (5)]. The dihedral angles between the two aromatic rings are 74.9 (1) and 71.0 (3)° in the two disorder components. The crystal structure features centrosymmetric dimers linked by pairs of N—H⋯O hydrogen bonds.

## Related literature

For the preparation of the title compound, see: Savitha & Gowda (2006 ▶). For our studies of the effect of substituents on the structures of *N*-(ar­yl)aryl­sulfonamides, see: Gowda *et al.* (2008 ▶, 2010**a* ▶,b* ▶); For related structures, see: Gelbrich *et al.* (2007 ▶); Perlovich *et al.* (2006 ▶).
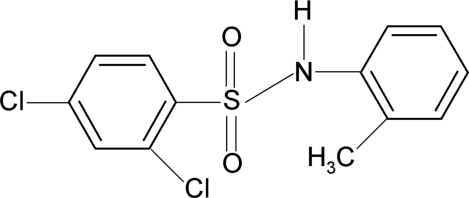


## Experimental

### 

#### Crystal data

C_13_H_11_Cl_2_NO_2_S
*M* *_r_* = 316.19
Monoclinic, 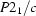

*a* = 8.106 (1) Å
*b* = 14.854 (2) Å
*c* = 11.772 (1) Å
β = 97.34 (1)°
*V* = 1405.8 (3) Å^3^
*Z* = 4
Cu *K*α radiation
μ = 5.52 mm^−1^
*T* = 299 K
0.35 × 0.25 × 0.25 mm

#### Data collection

Enraf–Nonius CAD-4 diffractometer
Absorption correction: multi-scan (North *et al.*, 1968 ▶) *T*_min_ = 0.248, *T*_max_ = 0.339
5256 measured reflections
2501 independent reflections
2205 reflections with *I* > 2σ(*I*)
*R*_int_ = 0.033
3 standard reflections every 120 min  intensity decay: 1.0%

#### Refinement

*R*[*F* ^2^ > 2σ(*F* ^2^)] = 0.039
*wR*(*F* ^2^) = 0.098
*S* = 1.04
2501 reflections
240 parameters
8 restraints
H atoms treated by a mixture of independent and constrained refinement
Δρ_max_ = 0.44 e Å^−3^
Δρ_min_ = −0.38 e Å^−3^

### 

Data collection: *CAD-4-PC* (Enraf–Nonius, 1996 ▶); cell refinement: *CAD-4-PC*; data reduction: *REDU4* (Stoe & Cie, 1987 ▶); program(s) used to solve structure: *SHELXS97* (Sheldrick, 2008 ▶); program(s) used to refine structure: *SHELXL97* (Sheldrick, 2008 ▶); molecular graphics: *PLATON* (Spek, 2009 ▶); software used to prepare material for publication: *SHELXL97*.

## Supplementary Material

Crystal structure: contains datablocks I, global. DOI: 10.1107/S1600536810035166/bt5340sup1.cif

Structure factors: contains datablocks I. DOI: 10.1107/S1600536810035166/bt5340Isup2.hkl

Additional supplementary materials:  crystallographic information; 3D view; checkCIF report

## Figures and Tables

**Table 1:** Hydrogen-bond geometry (Å, °)

*D*—H⋯*A*	*D*—H	H⋯*A*	*D*⋯*A*	*D*—H⋯*A*
N1—H1*N*⋯O2^i^	0.84 (2)	2.13 (2)	2.936 (2)	159 (2)

Symmetry code: (i) 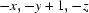.

**Fig. 1.**
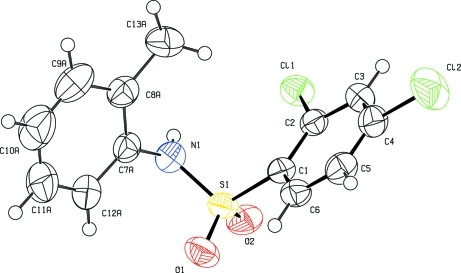
Molecular structure of (I), showing the atom labelling scheme and displacement ellipsoids are drawn at the 50% probability level. Only the major occupied component is shown.

**Fig. 2.**
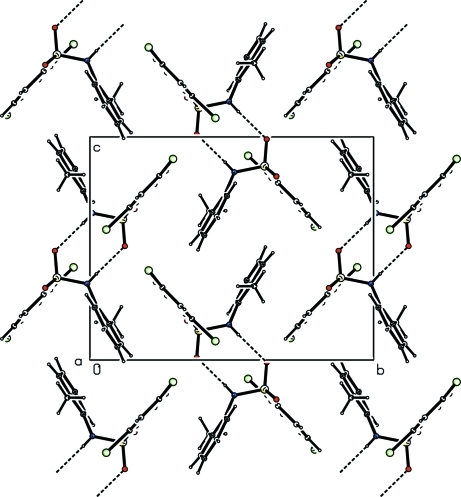
Molecular packing of (I) with hydrogen bonding shown as dashed lines.

## supplementary crystallographic information

### Comment

As part of a study of the substituent effects on the structures of *N*-(aryl)arylsulfonamides (Gowda *et al.*, 2008; Gowda *et al.* 2010**a*,b*), the structure of 2,4-dichloro-*N*-(2-methylphenyl)-benzenesulfonamide (I) has been determined (Fig. 1). The methylsubstituted anilino ring is disordered. The conformations of the N—C bonds in the C—SO_2_—NH—C segment have *gauche* torsions with respect to the S═O bonds in one of the disordered components.

The molecule is twisted at the S atom with the C1—SO_2_—NH—C7 torsion angles of -85.1 (3)° and -47.2 (5)° in the major and minor components, respectively, compared to the values of 55.1 (3)° (molecule 1) and -48.3 (3)° (molecule 2) in 2,4-dichloro-*N*-(phenyl)-benzenesulfonamide (II) (Gowda *et al.*, 2010*b*), -60.2 (2)° in 2,4-dichloro-*N*- (3-methylphenyl)benzenesulfonamide (III)(Gowda *et al.*, 2010*a*) and 72.0 (2)° in *N*-(2-methylphenyl)-benzenesulfonamide (IV) (Gowda *et al.*, 2008).

The sulfonyl benzene and the aniline benzene rings in (I) are tilted relative to each other by 74.9 (1)° and 71.0 (3)° in the two components, compared to the values of 80.5 (2)° in molecule 1 and 64.9 (1)° in molecule 2 of (II), 68.6 (1)° in (III) and 61.5 (1)° in (IV).

The other bond parameters in (I) are similar to those observed in (II), (III), (IV) and other aryl sulfonamides (Perlovich *et al.*, 2006; Gelbrich *et al.*, 2007).

In the crystal structure, the pairs of intermolecular N–H···O hydrogen bonds (Table 1) link the molecules through inversion-related dimers into infinite chains running parallel to the *c*-axis. Part of the crystal structure is shown in Fig. 2.

### Experimental

The solution of 1,3-dichlorobenzene (10 ml) in chloroform (40 ml) was treated dropwise with chlorosulfonic acid (25 ml) at 0 ° C. After the initial evolution of hydrogen chloride subsided, the reaction mixture was brought to room temperature and poured into crushed ice in a beaker. The chloroform layer was separated, washed with cold water and allowed to evaporate slowly. The residual 2,4-dichlorobenzenesulfonylchloride was treated with *o*-toluidine in the stoichiometric ratio and boiled for ten minutes. The reaction mixture was then cooled to room temperature and added to ice cold water (100 ml). The resultant solid 2,4-dichloro-*N*-(2-methylphenyl)benzenesulfonamide was filtered under suction and washed thoroughly with cold water. It was then recrystallized to constant melting point from dilute ethanol. The purity of the compound was checked and characterized by recording its infrared and NMR spectra (Savitha & Gowda, 2006).

Prism like colourless single crystals used in X-ray diffraction studies were grown in ethanolic solution by slow evaporation at room temperature.

### Refinement

The H atom of the NH group was located in a difference map and its coordinates were refined with the N—H distance restrained to 0.86 (2) %A. The other H atoms were positioned with idealized geometry using a riding model with C—H = 0.93–0.96 Å. All H atoms were refined with isotropic displacement parameters set to 1.2 times of the *U*_eq_ of the parent atom.

Atoms C7–C12 of the phenyl ring and C13 of the methyl group are disordered and were refined using a split model. The corresponding site-occupation factors were refined so that their sum was unity [0.705 (5) and 0.295 (5)]. The corresponding bond distances in the disordered groups were restrained to be equal.

### Crystal data

**Table:**

C_13_H_11_Cl_2_NO_2_S	*F*(000) = 648
*M**_r_* = 316.19	*D*_x_ = 1.494 Mg m^−^^3^
Monoclinic, *P*2_1_/*c*	Cu *K*α radiation, λ = 1.54180 Å
Hall symbol: -P 2ybc	Cell parameters from 25 reflections
*a* = 8.106 (1) Å	θ = 4.6–16.5°
*b* = 14.854 (2) Å	µ = 5.52 mm^−^^1^
*c* = 11.772 (1) Å	*T* = 299 K
β = 97.34 (1)°	Prism, colourless
*V* = 1405.8 (3) Å^3^	0.35 × 0.25 × 0.25 mm
*Z* = 4	

### Data collection

**Table:**

Enraf–Nonius CAD-4 diffractometer	2205 reflections with *I* > 2σ(*I*)
Radiation source: fine-focus sealed tube	*R*_int_ = 0.033
graphite	θ_max_ = 67.0°, θ_min_ = 4.8°
ω/2θ scans	*h* = 0→9
Absorption correction: multi-scan (North *et al.*, 1968)	*k* = −17→17
*T*_min_ = 0.248, *T*_max_ = 0.339	*l* = −14→13
5256 measured reflections	3 standard reflections every 120 min
2501 independent reflections	intensity decay: 1.0%

### Refinement

**Table:**

Refinement on *F*^2^	Secondary atom site location: difference Fourier map
Least-squares matrix: full	Hydrogen site location: inferred from neighbouring sites
*R*[*F*^2^ > 2σ(*F*^2^)] = 0.039	H atoms treated by a mixture of independent and constrained refinement
*wR*(*F*^2^) = 0.098	*w* = 1/[σ^2^(*F*_o_^2^) + (0.0521*P*)^2^ + 0.4627*P*] where *P* = (*F*_o_^2^ + 2*F*_c_^2^)/3
*S* = 1.04	(Δ/σ)_max_ = 0.005
2501 reflections	Δρ_max_ = 0.44 e Å^−^^3^
240 parameters	Δρ_min_ = −0.38 e Å^−^^3^
8 restraints	Extinction correction: *SHELXL97* (Sheldrick, 2008), Fc^*^=kFc[1+0.001xFc^2^λ^3^/sin(2θ)]^-1/4^
Primary atom site location: structure-invariant direct methods	Extinction coefficient: 0.0021 (3)

### Special details

**Table:**

Geometry. All e.s.d.'s (except the e.s.d. in the dihedral angle between two l.s. planes) are estimated using the full covariance matrix. The cell e.s.d.'s are taken into account individually in the estimation of e.s.d.'s in distances, angles and torsion angles; correlations between e.s.d.'s in cell parameters are only used when they are defined by crystal symmetry. An approximate (isotropic) treatment of cell e.s.d.'s is used for estimating e.s.d.'s involving l.s. planes.
Refinement. Refinement of *F*^2^ against ALL reflections. The weighted *R*-factor *wR* and goodness of fit *S* are based on *F*^2^, conventional *R*-factors *R* are based on *F*, with *F* set to zero for negative *F*^2^. The threshold expression of *F*^2^ > σ(*F*^2^) is used only for calculating *R*-factors(gt) *etc*. and is not relevant to the choice of reflections for refinement. *R*-factors based on *F*^2^ are statistically about twice as large as those based on *F*, and *R*- factors based on ALL data will be even larger.

### Fractional atomic coordinates and isotropic or equivalent isotropic displacement parameters (Å^2^)

**Table:**

	*x*	*y*	*z*	*U*_iso_*/*U*_eq_	Occ. (<1)
C1	0.1368 (2)	0.33703 (12)	0.20574 (15)	0.0386 (4)	
C2	0.2961 (3)	0.36013 (12)	0.18389 (16)	0.0404 (4)	
C3	0.4340 (3)	0.31932 (15)	0.24250 (19)	0.0502 (5)	
H3	0.5403	0.3342	0.2269	0.060*	
C4	0.4113 (3)	0.25585 (15)	0.3250 (2)	0.0533 (5)	
C5	0.2555 (3)	0.23232 (14)	0.34875 (19)	0.0520 (5)	
H5	0.2430	0.1899	0.4051	0.062*	
C6	0.1183 (3)	0.27211 (13)	0.28857 (17)	0.0450 (5)	
H6	0.0124	0.2556	0.3032	0.054*	
C7A	−0.1071 (5)	0.5236 (2)	0.2565 (3)	0.0446 (8)	0.705 (5)
C8A	0.0002 (5)	0.5685 (2)	0.3379 (4)	0.0532 (9)	0.705 (5)
C9A	−0.072 (2)	0.6089 (7)	0.4284 (7)	0.071 (3)	0.705 (5)
H9A	−0.0061	0.6448	0.4807	0.085*	0.705 (5)
C10A	−0.2337 (8)	0.5970 (3)	0.4412 (4)	0.0903 (16)	0.705 (5)
H10A	−0.2750	0.6187	0.5061	0.108*	0.705 (5)
C11A	−0.3369 (8)	0.5525 (4)	0.3577 (6)	0.0851 (17)	0.705 (5)
H11A	−0.4495	0.5469	0.3644	0.102*	0.705 (5)
C12A	−0.2749 (7)	0.5167 (3)	0.2651 (5)	0.0608 (12)	0.705 (5)
H12A	−0.3455	0.4877	0.2081	0.073*	0.705 (5)
C13A	0.1816 (13)	0.5795 (9)	0.3311 (9)	0.074 (3)	0.705 (5)
H13A	0.1967	0.6110	0.2619	0.088*	0.705 (5)
H13B	0.2331	0.5214	0.3309	0.088*	0.705 (5)
H13C	0.2316	0.6134	0.3960	0.088*	0.705 (5)
N1	−0.0460 (2)	0.49066 (12)	0.15829 (14)	0.0462 (4)	
H1N	0.003 (3)	0.5230 (16)	0.1141 (19)	0.055*	
O1	−0.1805 (2)	0.34262 (12)	0.17683 (16)	0.0629 (4)	
O2	−0.0363 (2)	0.37672 (10)	0.01067 (12)	0.0560 (4)	
Cl1	0.32844 (7)	0.44230 (4)	0.08450 (4)	0.05248 (18)	
Cl2	0.58543 (10)	0.20713 (6)	0.40131 (8)	0.0887 (3)	
S1	−0.04615 (6)	0.38428 (3)	0.13094 (4)	0.04322 (17)	
C7B	−0.0109 (13)	0.5386 (5)	0.2733 (7)	0.043 (2)	0.295 (5)
C8B	−0.1310 (11)	0.5537 (5)	0.3435 (6)	0.055 (2)	0.295 (5)
C9B	−0.080 (4)	0.5925 (14)	0.4520 (14)	0.052 (4)	0.295 (5)
H9B	−0.1519	0.5919	0.5073	0.062*	0.295 (5)
C10B	0.0727 (13)	0.6305 (6)	0.4773 (8)	0.068 (3)	0.295 (5)
H10B	0.0964	0.6653	0.5429	0.082*	0.295 (5)
C11B	0.1903 (13)	0.6177 (7)	0.4071 (10)	0.074 (3)	0.295 (5)
H11B	0.2974	0.6398	0.4274	0.088*	0.295 (5)
C12B	0.151 (3)	0.5715 (16)	0.3043 (19)	0.054 (4)	0.295 (5)
H12B	0.2317	0.5624	0.2561	0.064*	0.295 (5)
C13B	−0.3078 (15)	0.5262 (10)	0.3116 (11)	0.067 (4)	0.295 (5)
H13D	−0.3132	0.4623	0.2989	0.081*	0.295 (5)
H13E	−0.3532	0.5569	0.2429	0.081*	0.295 (5)
H13F	−0.3709	0.5415	0.3725	0.081*	0.295 (5)

### Atomic displacement parameters (Å^2^)

**Table:**

	*U*^11^	*U*^22^	*U*^33^	*U*^12^	*U*^13^	*U*^23^
C1	0.0406 (10)	0.0357 (9)	0.0390 (9)	−0.0031 (8)	0.0029 (7)	−0.0023 (7)
C2	0.0429 (11)	0.0354 (9)	0.0432 (10)	−0.0030 (8)	0.0065 (8)	−0.0020 (8)
C3	0.0395 (11)	0.0490 (12)	0.0620 (13)	−0.0002 (9)	0.0059 (9)	−0.0007 (10)
C4	0.0516 (13)	0.0457 (11)	0.0600 (12)	0.0073 (10)	−0.0036 (10)	0.0029 (10)
C5	0.0623 (14)	0.0420 (10)	0.0511 (11)	−0.0001 (10)	0.0051 (10)	0.0077 (9)
C6	0.0460 (11)	0.0422 (10)	0.0474 (11)	−0.0066 (9)	0.0082 (8)	0.0013 (8)
C7A	0.050 (2)	0.0363 (16)	0.0468 (19)	0.0050 (15)	0.0028 (16)	0.0013 (13)
C8A	0.060 (3)	0.0448 (18)	0.053 (2)	0.0001 (16)	0.0025 (18)	0.0001 (16)
C9A	0.111 (5)	0.051 (5)	0.050 (4)	−0.003 (3)	0.012 (4)	−0.002 (4)
C10A	0.110 (4)	0.084 (3)	0.085 (3)	0.007 (3)	0.043 (3)	−0.020 (2)
C11A	0.071 (3)	0.084 (4)	0.109 (5)	0.005 (3)	0.047 (3)	−0.022 (3)
C12A	0.056 (3)	0.053 (2)	0.074 (4)	0.0074 (18)	0.013 (2)	−0.007 (2)
C13A	0.059 (4)	0.080 (5)	0.077 (7)	−0.011 (3)	−0.006 (4)	−0.019 (4)
N1	0.0526 (11)	0.0438 (9)	0.0424 (9)	0.0006 (8)	0.0069 (7)	0.0035 (7)
O1	0.0401 (9)	0.0595 (9)	0.0888 (12)	−0.0069 (7)	0.0073 (8)	0.0146 (8)
O2	0.0650 (11)	0.0557 (9)	0.0431 (8)	−0.0070 (7)	−0.0094 (7)	−0.0048 (6)
Cl1	0.0507 (3)	0.0533 (3)	0.0544 (3)	−0.0078 (2)	0.0107 (2)	0.0094 (2)
Cl2	0.0639 (4)	0.0861 (5)	0.1097 (6)	0.0179 (4)	−0.0134 (4)	0.0310 (4)
S1	0.0373 (3)	0.0435 (3)	0.0472 (3)	−0.00531 (19)	−0.00115 (19)	0.00201 (19)
C7B	0.062 (7)	0.032 (4)	0.033 (4)	0.002 (4)	−0.001 (4)	−0.008 (3)
C8B	0.068 (6)	0.045 (4)	0.054 (5)	0.007 (4)	0.017 (4)	−0.002 (3)
C9B	0.091 (10)	0.035 (7)	0.033 (6)	0.004 (6)	0.022 (6)	0.007 (5)
C10B	0.093 (7)	0.053 (5)	0.057 (5)	0.004 (5)	0.004 (5)	−0.020 (4)
C11B	0.064 (6)	0.075 (6)	0.076 (6)	−0.003 (5)	−0.014 (5)	−0.030 (5)
C12B	0.065 (11)	0.046 (6)	0.047 (7)	−0.006 (6)	−0.004 (7)	−0.014 (5)
C13B	0.048 (8)	0.088 (10)	0.069 (10)	−0.002 (6)	0.021 (6)	−0.021 (7)

### Geometric parameters (Å, °)

**Table:**

C1—C2	1.391 (3)	C12A—H12A	0.9300
C1—C6	1.393 (3)	C13A—H13A	0.9600
C1—S1	1.770 (2)	C13A—H13B	0.9600
C2—C3	1.377 (3)	C13A—H13C	0.9600
C2—Cl1	1.7337 (19)	N1—C7B	1.524 (9)
C3—C4	1.382 (3)	N1—S1	1.6125 (18)
C3—H3	0.9300	N1—H1N	0.844 (17)
C4—C5	1.373 (3)	O1—S1	1.4183 (17)
C4—Cl2	1.732 (2)	O2—S1	1.4326 (15)
C5—C6	1.373 (3)	C7B—C8B	1.374 (11)
C5—H5	0.9300	C7B—C12B	1.402 (17)
C6—H6	0.9300	C8B—C9B	1.415 (16)
C7A—C12A	1.381 (7)	C8B—C13B	1.492 (13)
C7A—C8A	1.381 (5)	C9B—C10B	1.36 (3)
C7A—N1	1.403 (4)	C9B—H9B	0.9300
C8A—C9A	1.413 (11)	C10B—C11B	1.352 (15)
C8A—C13A	1.492 (11)	C10B—H10B	0.9300
C9A—C10A	1.350 (15)	C11B—C12B	1.393 (16)
C9A—H9A	0.9300	C11B—H11B	0.9300
C10A—C11A	1.375 (8)	C12B—H12B	0.9300
C10A—H10A	0.9300	C13B—H13D	0.9600
C11A—C12A	1.366 (7)	C13B—H13E	0.9600
C11A—H11A	0.9300	C13B—H13F	0.9600
			
C2—C1—C6	119.02 (18)	C8A—C13A—H13C	109.5
C2—C1—S1	123.26 (14)	H13A—C13A—H13C	109.5
C6—C1—S1	117.69 (15)	H13B—C13A—H13C	109.5
C3—C2—C1	120.80 (19)	C7A—N1—C7B	31.8 (3)
C3—C2—Cl1	117.70 (16)	C7A—N1—S1	121.00 (19)
C1—C2—Cl1	121.49 (15)	C7B—N1—S1	129.1 (3)
C2—C3—C4	118.6 (2)	C7A—N1—H1N	123.6 (17)
C2—C3—H3	120.7	C7B—N1—H1N	103.4 (17)
C4—C3—H3	120.7	S1—N1—H1N	115.0 (17)
C5—C4—C3	121.7 (2)	O1—S1—O2	118.99 (10)
C5—C4—Cl2	119.84 (18)	O1—S1—N1	109.42 (11)
C3—C4—Cl2	118.43 (18)	O2—S1—N1	105.99 (9)
C4—C5—C6	119.4 (2)	O1—S1—C1	105.83 (10)
C4—C5—H5	120.3	O2—S1—C1	108.16 (10)
C6—C5—H5	120.3	N1—S1—C1	108.06 (9)
C5—C6—C1	120.5 (2)	C8B—C7B—C12B	119.8 (13)
C5—C6—H6	119.8	C8B—C7B—N1	123.0 (9)
C1—C6—H6	119.8	C12B—C7B—N1	117.1 (11)
C12A—C7A—C8A	121.6 (4)	C7B—C8B—C9B	117.4 (14)
C12A—C7A—N1	119.3 (4)	C7B—C8B—C13B	122.4 (9)
C8A—C7A—N1	118.9 (4)	C9B—C8B—C13B	120.1 (14)
C7A—C8A—C9A	116.3 (7)	C10B—C9B—C8B	121.5 (16)
C7A—C8A—C13A	123.6 (5)	C10B—C9B—H9B	119.2
C9A—C8A—C13A	120.0 (7)	C8B—C9B—H9B	119.2
C10A—C9A—C8A	122.0 (8)	C11B—C10B—C9B	120.1 (10)
C10A—C9A—H9A	119.0	C11B—C10B—H10B	120.0
C8A—C9A—H9A	119.0	C9B—C10B—H10B	120.0
C9A—C10A—C11A	119.6 (5)	C10B—C11B—C12B	119.9 (13)
C9A—C10A—H10A	120.2	C10B—C11B—H11B	120.1
C11A—C10A—H10A	120.2	C12B—C11B—H11B	120.1
C12A—C11A—C10A	120.4 (6)	C11B—C12B—C7B	120.1 (18)
C12A—C11A—H11A	119.8	C11B—C12B—H12B	120.0
C10A—C11A—H11A	119.8	C7B—C12B—H12B	120.0
C11A—C12A—C7A	119.7 (6)	C8B—C13B—H13D	109.5
C11A—C12A—H12A	120.1	C8B—C13B—H13E	109.5
C7A—C12A—H12A	120.1	H13D—C13B—H13E	109.5
C8A—C13A—H13A	109.5	C8B—C13B—H13F	109.5
C8A—C13A—H13B	109.5	H13D—C13B—H13F	109.5
H13A—C13A—H13B	109.5	H13E—C13B—H13F	109.5
			
C6—C1—C2—C3	0.4 (3)	C7A—N1—S1—O1	29.7 (3)
S1—C1—C2—C3	−178.13 (16)	C7B—N1—S1—O1	67.6 (5)
C6—C1—C2—Cl1	−178.41 (15)	C7A—N1—S1—O2	159.2 (2)
S1—C1—C2—Cl1	3.1 (2)	C7B—N1—S1—O2	−162.9 (5)
C1—C2—C3—C4	−1.0 (3)	C7A—N1—S1—C1	−85.1 (2)
Cl1—C2—C3—C4	177.79 (16)	C7B—N1—S1—C1	−47.2 (5)
C2—C3—C4—C5	0.5 (3)	C2—C1—S1—O1	179.13 (16)
C2—C3—C4—Cl2	−178.15 (17)	C6—C1—S1—O1	0.62 (18)
C3—C4—C5—C6	0.6 (3)	C2—C1—S1—O2	50.59 (18)
Cl2—C4—C5—C6	179.28 (17)	C6—C1—S1—O2	−127.93 (15)
C4—C5—C6—C1	−1.3 (3)	C2—C1—S1—N1	−63.73 (18)
C2—C1—C6—C5	0.8 (3)	C6—C1—S1—N1	117.75 (16)
S1—C1—C6—C5	179.39 (16)	C7A—N1—C7B—C8B	1.1 (5)
C12A—C7A—C8A—C9A	−1.7 (7)	S1—N1—C7B—C8B	−86.0 (8)
N1—C7A—C8A—C9A	173.1 (5)	C7A—N1—C7B—C12B	−176.0 (15)
C12A—C7A—C8A—C13A	−178.6 (8)	S1—N1—C7B—C12B	96.9 (13)
N1—C7A—C8A—C13A	−3.8 (8)	C12B—C7B—C8B—C9B	−7.9 (19)
C7A—C8A—C9A—C10A	6.4 (11)	N1—C7B—C8B—C9B	175.0 (12)
C13A—C8A—C9A—C10A	−176.6 (9)	C12B—C7B—C8B—C13B	175.7 (15)
C8A—C9A—C10A—C11A	−7.2 (13)	N1—C7B—C8B—C13B	−1.4 (12)
C9A—C10A—C11A—C12A	3.2 (11)	C7B—C8B—C9B—C10B	14 (3)
C10A—C11A—C12A—C7A	1.3 (9)	C13B—C8B—C9B—C10B	−170.0 (16)
C8A—C7A—C12A—C11A	−2.0 (7)	C8B—C9B—C10B—C11B	−12 (3)
N1—C7A—C12A—C11A	−176.8 (5)	C9B—C10B—C11B—C12B	5(2)
C12A—C7A—N1—C7B	177.2 (8)	C10B—C11B—C12B—C7B	0(3)
C8A—C7A—N1—C7B	2.3 (5)	C8B—C7B—C12B—C11B	1(2)
C12A—C7A—N1—S1	−67.5 (4)	N1—C7B—C12B—C11B	178.6 (13)
C8A—C7A—N1—S1	117.6 (3)		

### Hydrogen-bond geometry (Å, °)

**Table:**

*D*—H···*A*	*D*—H	H···*A*	*D*···*A*	*D*—H···*A*
N1—H1N···O2^i^	0.84 (2)	2.13 (2)	2.936 (2)	159 (2)

Symmetry codes: (i) −*x*, −*y*+1, −*z*.
